# Comparative analysis of osteoclast function in symptomatic and asymptomatic individuals with cherubism-causing SH3BP2 mutation

**DOI:** 10.1093/jbmrpl/ziaf148

**Published:** 2025-09-09

**Authors:** Chen Abramovitch-Dahan, Svetlana Katchkovsky, Yuval Zur, Gal Gozlan, Nitsan Nimni, Eitan Bar Droma, Navot Givol, Alex Geftler, Merav Fraenkel, Anat Reiner-Benaim, Kent Søe, Noam Levaot

**Affiliations:** Department of Physiology and Cell Biology, Faculty of Health Sciences, Ben-Gurion University of the Negev, Beer-Sheva, 8410500, Israel; Department of Physiology and Cell Biology, Faculty of Health Sciences, Ben-Gurion University of the Negev, Beer-Sheva, 8410500, Israel; Avram and Stella Goldstein-Goren Department of Biotechnology Engineering, Ben-Gurion University of the Negev, Beer-Sheva, 8410500, Israel; Department of Physiology and Cell Biology, Faculty of Health Sciences, Ben-Gurion University of the Negev, Beer-Sheva, 8410500, Israel; Department of Physiology and Cell Biology, Faculty of Health Sciences, Ben-Gurion University of the Negev, Beer-Sheva, 8410500, Israel; Oral and Maxillofacial Surgery Unit, Soroka Medical Center, Beer-Sheva, 8410101, Israel; Oral and Maxillofacial Surgery Unit, Soroka Medical Center, Beer-Sheva, 8410101, Israel; Orthopedic Surgery Department, Soroka Medical Center, Beer-Sheva, 8457108, Israel; Endocrinology Unit, Ben-Gurion University and Soroka Medical Center, Beer-Sheva, 8410101, Israel; Department of Epidemiology, Biostatistics and Community Health Sciences, Faculty of Health Sciences, School of Public Health, Ben-Gurion University of the Negev, Beer-Sheba, 8410500, Israel; Clinical Cell Biology, Pathology Research Unit, Department of Clinical Research, University of Southern Denmark, 5230, Odense, Denmark; Department of Pathology, Odense University Hospital, Odense, 5230, Denmark; Department of Physiology and Cell Biology, Faculty of Health Sciences, Ben-Gurion University of the Negev, Beer-Sheva, 8410500, Israel; Regenerative Medicine and Stem Cell Research Center, Ben-Gurion University of the Negev, Beer-Sheva, 8410500, Israel

**Keywords:** cherubism, osteoclast, SH3BP2, granuloma, bone resorption

## Abstract

Cherubism is a rare autosomal dominant bone disease of the maxilla and mandible with variable severity. Most patients harbor a mutation in SH3 domain-binding protein 2 (SH3BP2), yet factors influencing genetic penetrance and clinical severity remain unclear. In mice, this mutation induces tumor necrosis factor alpha (TNF-α)-mediated systemic inflammation, though its role in human cherubism is debated. Multinucleated osteoclasts (OCs), rather than macrophages, are linked to symptom severity, but whether this results from progenitor differentiation or environmental factors is unknown. To elucidate this, OC differentiation and resorption were compared in PBMCs from two symptomatic and one asymptomatic carrier of the same cherubism mutation. All carriers exhibited larger OCs than healthy controls when cultured with RANKL or TNF-α. On bone slices, OCs from carriers resorbed more bone than controls, with TNF-α exerting a weaker effect than RANKL. No significant differences were observed between symptomatic and asymptomatic carriers, suggesting that symptom severity is influenced by microenvironmental factors external to OCs. Additionally, while TNF-α promotes giant cell formation in cherubism OCs, its impact on resorption is limited. These findings may explain why TNF-α inhibition reduces giant cell numbers in cherubism lesions without improving clinical outcomes.

## Graphical Abstract

**Graphical Abstract:**
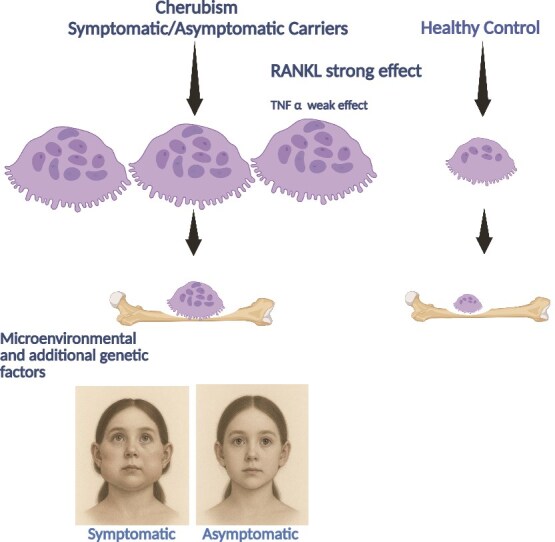

## Introduction

Cherubism is a rare autosomal dominant bone disorder characterized by symmetrical fibrotic bone lesions in the maxilla and mandible.^1^ Clinical symptoms of cherubism patients appear between 2 and 5 yr of age, progress until puberty, and then usually regress in adulthood.^2^ The clinical symptoms include: swelling and expansion of the affected bones, destruction of cortical bones, tooth displacement, and tooth agenesis. The severity of the clinical symptoms is variable and could range from asymptomatic bilateral swelling in the jaws to life threatening bone lesions.^3–5^ Although much knowledge has been gained on the underlying pathological mechanisms driving cherubism, the factors that determine the appearance of symptoms and their severity are still unknown.

Histologically, cherubism granulomas are filled with a dense fibrotic tissue and are rich in multinucleated giant cells (MGCs), some of which are in contact with the bone and others are scattered throughout the fibrotic tissue.^6^

Cherubism is caused by mutations in the gene encoding the adaptor protein SH3 domain-binding protein 2 (SH3BP2).^7^ These mutations can be inherited or occur sporadically.^8^ In most cases of cherubism, the mutations are missense mutations clustered within a hexapeptide with the amino acid sequence RSPPDG located at position 415-420 in the protein.^7^ The most common mutation is c.1244G>A resulting in p.R415Q in both familial and sporadic cases.^8^ SH3BP2 is an adaptor protein that coordinates signals from integrins, c-fms, Itams, and the RANK receptor with downstream activation of Src, Syk, and Vav-family protein kinases followed by activation of the osteoclast (OC) master transcription factor NFATc1.^9–13^ The causative role for SH3BP2 in cherubism has been confirmed in a mouse model harboring a P416R knock-in mutation (KI) in murine SH3BP2 (equivalent to P418R in humans). Mice homozygous for the mutation show extensive bone resorption and recapitulate the bone lesions in the maxilla and mandible seen in cherubism patients.^14^ We showed that SH3BP2 serves as a substrate for the poly-ADP-ribosyltransferases Tankyrase 1 and 2. Tankyrases bind SH3BP2 and poly-ADP-ribosylate it (Parsylate). Parsylation of SH3BP2 is followed by ubiquitination by the E3 ligase RNF146 and subsequent proteasomal degradation. Each of the mutations in the SH3BP2 hexapeptide prevent Tankyrases binding, which is followed by the stabilization of SH3BP2 and enhanced signaling cascade involving Src and Syk kinases. These changes result in hyperactive and aggressive OCs with enhanced bone resorption.^15^ Unlike human SH3BP2 heterozygotes, heterozygous mice do not exhibit any cherubism phenotype. The homozygous KI mouse has high circulating tumor necrosis factor alpha (TNF-α) concentrations and suffer from systemic inflammation with macrophage infiltration into tissues. This systemic inflammation is rescued when SH3BP2 KI mice are crossed with mice deficient in either TNF-α or MYD88 (a mediator of toll-like receptor signaling) indicating that cherubism is an auto-inflammatory bone disease.^14^^,^^16^ Recently, it was shown that there is a correlation between the number of MGCs expressing OC markers and the severity of the bone lesions.^17^ However, it is not known if the increased OC numbers and the severity of the bone lesions in cherubism is a result of an increased differentiation potential of OC precursors or increased local osteoclastogenic signals.

To address this question, we compared the differentiation and bone resorption potential of OCs derived from peripheral blood progenitors of symptomatic and asymptomatic carriers of the SH3BP2 P418R mutation.

## Materials and methods

### Ethics statement

This study was carried out according to protocols approved by the Soroka Medical Center (No. 0166-17 SOR). Informed consent from all donors was documented in a written form. In case, the donor was minor, parental consent was obtained along with the participant’s assent.

Study population and samples. The cells described in the study were derived from blood donated by two sisters, aged 18 and 21 yr, that were diagnosed with cherubism. The diagnosis was confirmed by a genetic examination, which showed a P418R mutation in SH3BP2. Cells were also isolated from the blood of the 50-yr-old father, who was found to be an asymptomatic carrier of this mutation. For control blood from healthy donors, a total of 12 donors were used: four men (50-yr-old) and 8 women (ages 15, 18, 18, 18, 19, 21, 25, and 26) were used. Healthy donors were nonsmoking and under no medication. Experimental replicates included fresh samples.

### In vitro generation of human OCs

Peripheral blood mononuclear cells (PBMCs) were isolated from peripheral blood samples and differentiated to mature OCs as follow: whole blood samples were collected to CPT tubes and mononuclear cells were separated according to the manufacture protocol (BD Vacutainer, 362782). Samples were taken once from each donor and results from all donors were integrated across multiple runs. To induce RANK expression, 2.5 × 10^7^ PBMCs were seeded in T75 culture flasks and supplemented with 25 ng/mL M-CSF (R&D systems, 216-MC) for 3 d. The cells were then detached from the flask using Accutase (Sigma, A6964), counted, and re-seeded in 96-well plates for either differentiation or bone resorption assays (see “OC differentiation assay”).

### OC differentiation assay

To assess OC differentiation, 7.5 × 10^4^ cells from PBMCs cells pre-incubated with M-CSF were cultured in 96-well plate with differentiation medium (αMEM; M8042 sigma, 10% FBS, 5% Penstrep, and l-Glu, BI) supplemented with 25 ng/mL M-CSF and either 25 ng/mL RANKL (R&D systems, 390-TN) or TNF-α 100 ng/mL (R&D systems, 210-TA). Differentiation medium was changed after 2 d and after 5 d before the cells were fixed. Cells were fixed with 4% PFA for 10 min at room temperature washed three times with PBS and then stained using a TRAP staining kit (Sigma-Aldrich, 387A-1KT) according to the manufacturer’s protocol with additional staining of the nuclei with 4′,6-diamidino-2-phenylindole (DAPI). Osteoclast parameters were obtained by analysis of three wells from each individual and treatment. The OCs were observed with an Olympus ×83 microscope with an automated stage at 20x magnification. To sample each well evenly, we used a fixed diagram to define the same fields of view obtaining 20 images per well. A total of 1380 field of views were analyzed, including 5728 OCs and 70 846 nuclei within these OCs, for RANKL treatment, and a total of 660 field views were analyzed for 3716 OCs and 36 042 nuclei in OCs for TNF-α treatment.

Osteoclasts were defined as TRAP-positive cells harboring three or more nuclei and were counted in a double blind manner (the source of the sample: carrier or control and treatment RANKL or TNF-α were not known to the person performing the analysis), and the number of nuclei in the OCs per well (the sum of nuclei within OCs) and the total OC surface area were determined using ImageJ software by manually tracing individual OCs perimeters and manual counts of the nuclei with in these objects using the DAPI channel.

### Bone resorption assays

To assess bone resorption, PBMCs were cultured for three days in the presence of M-CSF (25 ng/mL) and then an equal number of PBMCs (1.8 × 10^5^ cells) were seeded on bovine cortical bone slices (BoneSlices.com), supplemented with M-CSF (25 ng/mL) and RANKL (25 ng/mL) or TNF-α 100 ng/mL. Media was changed every 2-3 d. After 12 d, the bone slices were stained with toluidine blue staining (Sigma, T3260) as was conducted by Vesprey and Yang.^18^ At the end of the resorption experiment, the bone slices were imaged using Olympus ×83 microscope. The whole bone slice was analyzed by generating a panoramic view of the slice using stitching of images taken with a 20x magnification. The eroded surface perimeter was manually traced and divided by the total surface of the slice. For each individual donor and treatment, 3-5 bone slices were used.

### Statistical analysis

**Figure 1:**
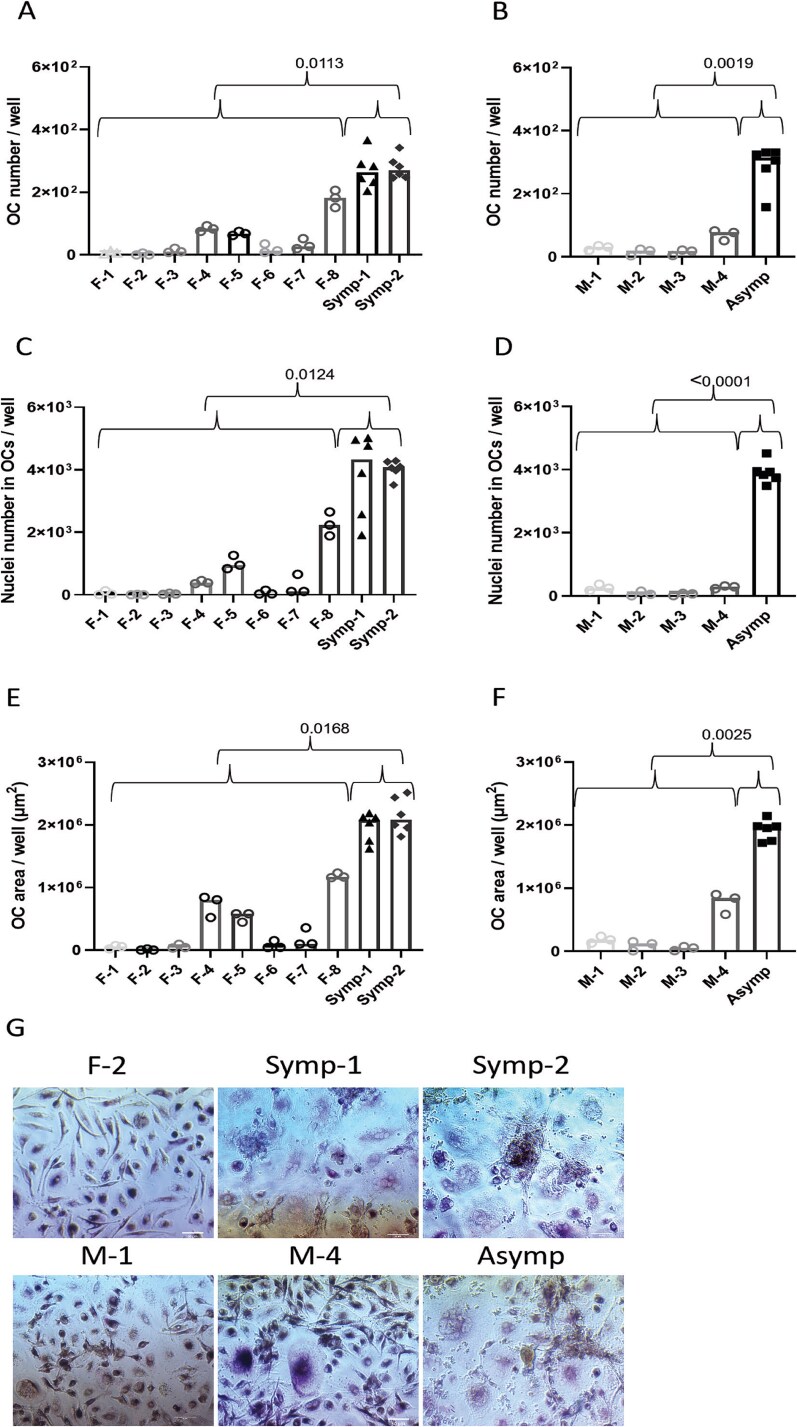
Osteoclasts from PBMCs of cherubism carriers, driven by RANKL, are more frequent and larger than OCs in healthy controls. Peripheral blood mononuclear cells were cultured in OC differentiation medium in the presence of M-CSF and RANKL. Cells were fixed and stained for TRAP and DAPI. (A and B) Total number of OCs per well, (C and D), total number of nuclei in OCs per well, and (E and F) total area occupied by OCs (μm^2^) per well. (G) Representative pictures of the different samples scale bar in length is 50 μm. Data are median of *n* = 3 or 6. A total of 60-120 frames were analyzed. A, C, and E are female samples and B, D, and F are male samples. F, M, Symp and Asymp refer to female, male, symptomatic and asymptomatic, respectively. Groups and treatments were compared using multivariate generalized linear mixed effects model. *p*-values that are presented are based on comparisons between carriers (Symp-1 and Symp-2 for females or Asymp for males) vs age and gender matched controls.

Adjusted effects of disease (carriers vs healthy controls) and type of treatment (RANKL vs TNF-α) were obtained by fitting a multivariate generalized linear mixed effects model for each measure (OCs number, nuclei number, OCs area, and ES), with the measure as a dependent variable, and group and treatment as dependent variables. An interaction effect between group and treatment was included to assess differences in effects between subgroups. The Gamma family with the log link function was used for the model, thereby generating means ratios as effects. The Wald method was used for calculating confidence intervals for the means ratios. A similar model was fit for each measure on the carriers only, to obtain the effects of symptoms (symptomatic vs asymptomatic). The 0.05 level was used for significance. The R statistical environment,^19^ including the lme4 package^20^ was used for analysis. Despite potential added variance due to multiple batches among the controls females, statistically significant differences were still found between the symptomatic and the controls. Accordingly, the plots (eg, Figure 1A, C, and  E), show the small extra variance added, compared to the substantial differences between the two groups.

## Results

In humans, physiological conditions characterized by enhanced bone resorption in vivo are reflected by enhanced fusion and bone resorption by OCs derived from blood monocytes.^21^^,^^22^ The differentiation potential and characteristics of PBMCs from human cherubism patients has not been studied before. For this purpose, we compared OC differentiation when using PBMCs isolated from three cherubism mutation-carriers (two symptomatic and one asymptomatic) to PBMCs isolated from gender and aged matched controls. The cherubism mutation-carriers were two sisters 18 and 21 yr old (ie, Symp-1 and Symp-2, respectively) at in an active phase of jaw lesions at and before treatment at the time of the donation. Both were diagnosed with cherubism and both exhibited classical symptoms like lesions in both jaws, mandibular dentition loss, hyperplastic appearance of the molars, and mandibular rami and body. Symp-1 has exhibited lesions in both jaws, causing a hyperplastic appearance of the molars and mandibular rami and body; while Symp-2 had lost most of her mandibular dentition, presenting with only 12 remaining teeth intraorally. A detailed account of clinical, radiological, and histological information for these specific patients was described in a previous publication.^23^ The father (ie, Asymp) was 50-yr-old at the time of the donation. He was genetically diagnosed as a cherubism mutation carrier but was not diagnosed with any clinical symptoms at present or in the past. His orthopantomogram shows extensive dental rehabilitation with implant supported bridges (Figure S1). There is no documented history of jaw lesions, periodontal disease, tooth agenesis, congenital dental anomalies, or any other explanation for the extent of dental reconstruction. There are no signs or indications of current cherubism-related lesions or other abnormalities in the mandibular bone, aside from two impacted mandibular wisdom teeth. The father has no comorbidities and is not on any medication, and his parents (the probands’ grandparents) had no history suggestive of cherubism. For gender and age matched controls, eight women ages 15, 18, 25, and 26 (ie, F1-8, respectively) and four 50-yr-old men (ie, M1-4) were recruited.

Peripheral blood mononuclear cells from all of the donors were cultured for 72 h in the presence of macrophage colony stimulating factor (M-CSF), then transferred and cultured in the presence of M-CSF and RANKL to induce OC differentiation. In cultures of PBMCs from the symptomatic and asymptomatic cherubism mutation-carriers, multinucleated cells appeared and reached maximal size 5 d after RANKL-addition. At this early stage, multinucleated cells were only starting to appear in most of the control cultures. Therefore, we terminated the experiment 5 d after RANKL-addition in both the experiment and control groups. Cells were defined as OCs when they were tartrate resistant acid phosphatase (TRAP) positive and contained three or more nuclei.

The number of OCs, the total number of nuclei within OCs, and the total area of OCs were manually quantified. Analysis of OC morphological parameters showed similar OC number (Figure 1A and B), nuclei number within OCs (*p*-value = 0.9243, Figure 1C and D), and total OC area (*p*-value = 0.1520, Figure 1E and F) between cultures of the two symptomatic cherubism mutation carriers and the asymptomatic carrier. When compared to the age and gender matched controls, both the symptomatic and the asymptomatic cell cultures had higher OC number (Figure 1A and B), nuclei (Figure 1C and D), and area (Figure 1E and F) Representative images are presented in Figure 1G. The statistical differences are summarized in Table 1.

**Table 1:** Statistical comparison between OC parameters of cherubism mutation—carriers vs matched healthy controls after RANKL treatment.

	**Symptomatic vs matched controls**		**Asymptomatic vs matched controls**	
**Parameter**	**Means ratio (95% CI)**	***p*-value**	**Means ratio (95% CI)**	***p*-value**
**OC number/well**	10.3 (1.7, 62.7)	0.0113	8.3 (1.9, 36.6)	0.0051
**Nuclei number/well**	26 (2, 336)	0.0124	23.5 (6.2, 88.9)	<0.0001
**OC area/well**	14.5 (1.6, 129.4)	0.0168	7 (2, 24.6)	0.0025
**Eroded surface/BS**	7.1 (2.5, 20)	0.0002	7.6 (1.2, 49.1)	0.0340

Mean ratio, that is, carriers vs gender and age matched controls. Groups and treatments were compared using multivariate generalized linear mixed effects model. *p*-values that are presented are based on comparisons between carriers (Symp-1 and Symp-2 for females or Asymp for males) vs age and gender matched controls.

Abbreviation: CI, confidence interval.

We next asked if OCs derived from PBMCs of cherubism mutation carriers, both symptomatic and asymptomatic, are more aggressive when it comes to bone resorption than the age and gender matched controls. For this purpose, we compared the bone resorption activity of their OCs in vitro.

**Figure 2:**
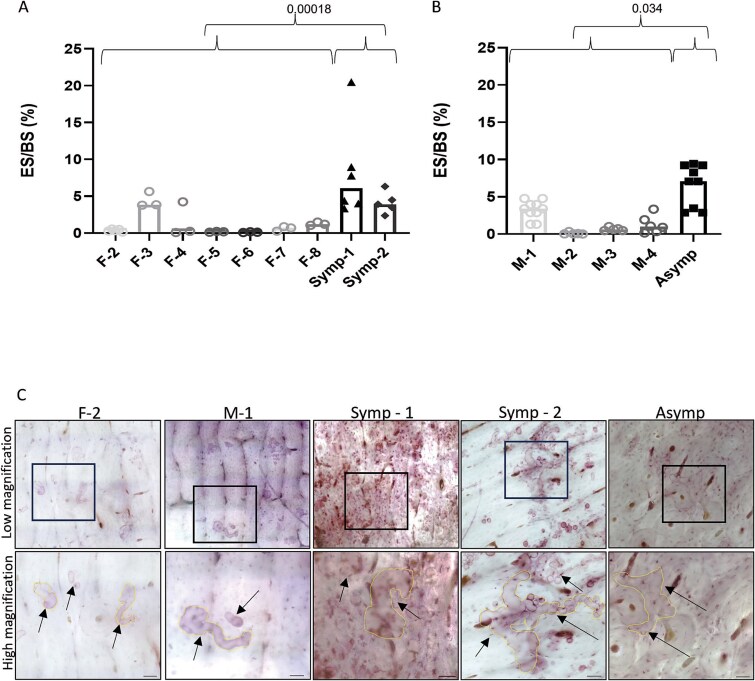
Osteoclasts generated from PBMCs of cherubism carriers, obtained through RANKL-mediated differentiation, resorb more bone than OCs from healthy controls. Human PBMCs were cultured in OC differentiation medium M-CSF and RANKL (25 ng/mL each) for 14 d (A-C). (A and B) The percentage of eroded surface (ES) per total bone surface (BS). Female samples (A) male samples (B). Groups and treatments were compared using multivariate generalized linear mixed effects model. *p*-values that are presented are comparisons between carriers (Symp-1 and Symp-2 for females or Asymp for males) vs age and gender matched controls. Images showing the representative largest trenches that were found in each group (low and high magnification) (C) F-1; 670 μm^2^ on average, M-1; 2041 μm^2^, Symp-1; 4045 μm^2^, Symp-2; 7616 μm^2^, asymptomatic; 3039 μm^2^. Arrows marking the ES and bright lines are marking the borders of the trenches’ area. F, M, Symp and Asymp refer to female, male, symptomatic and asymptomatic, respectively. Brightness and contrast were adjusted to gain a better visualization of ES, scale bar length is 50 μm.

On average, bone resorption areas of OCs derived from both the symptomatic (Figure 2A) and asymptomatic (Figure 2B). Cherubism mutation-carriers were more than seven-fold higher than the resorption areas of the OCs from the healthy controls (Table 1). Examination of the eroded surfaces revealed that some of the resorption trenches, formed by OCs from symptomatic and asymptomatic patients, were unique in their size and such trenches were not observed in the control samples (Figure 2C).

**Figure 3:**
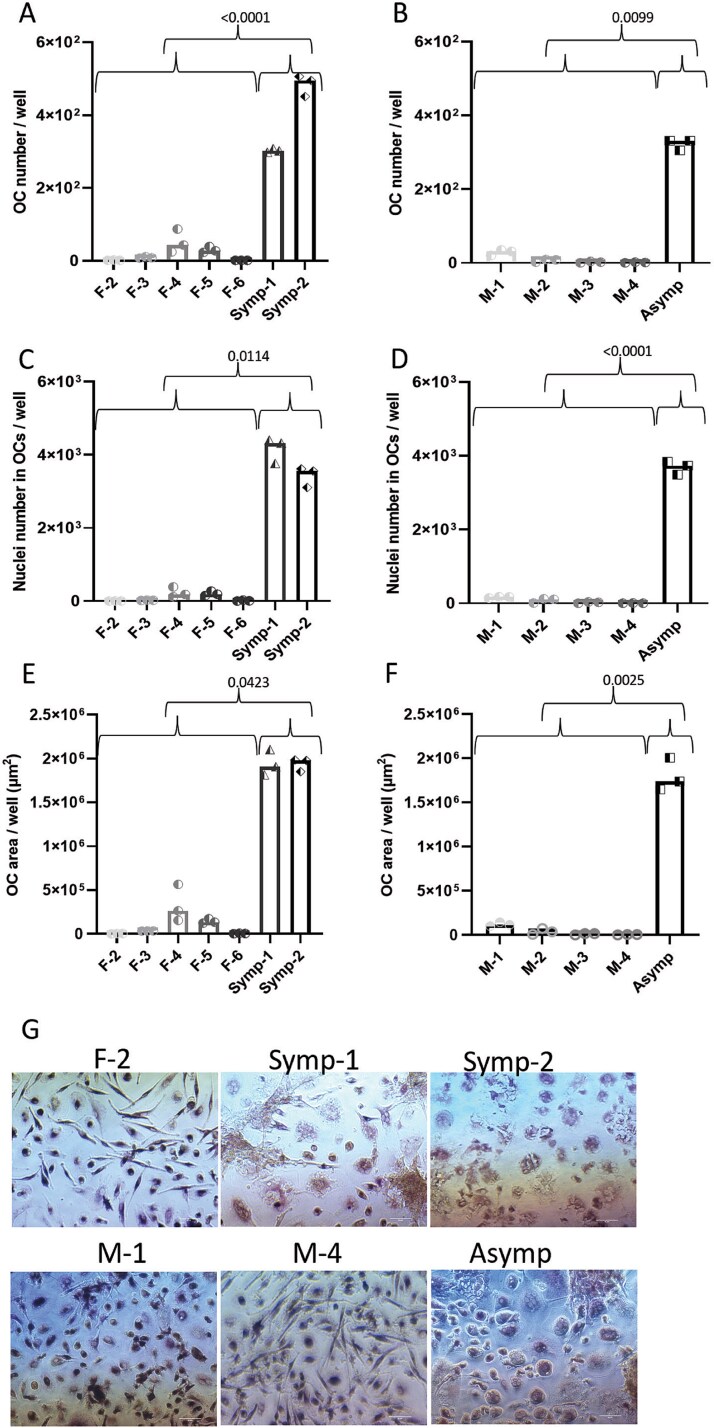
Osteoclasts from PBMCs of cherubism carriers, driven by TNF-α, are more frequent and larger than those from healthy donors. Human PBMC’s were cultured in osteoclast (OC) differentiation medium in the presence of M-CSF (25 ng/mL) and TNF-α (100 ng/mL). Cells were stained for TRAP and DAPI. (A and B) number of OCs per well, (C and D) number of nuclei in OCs per well, and (E and F) average OC area per well. (G) Representative pictures of the different samples, scale bar in length is 50 μm. Data are median of *n* = 3 or 6. A total of 60-120 frames were analyzed. F, M, Symp and Asymp refer to female, male, symptomatic and asymptomatic, respectively. Female samples (A, C, and E) and male samples (B, D, and F). (G) Representative pictures of the different samples scale bar in length is 50 μm. Groups and treatments were compared using multivariate generalized linear mixed effects model. *p*-values that are presented are based on comparisons between carriers (Symp-1 and Symp-2 for females or Asymp for males) vs age and gender matched controls. Abbreviation: OCs, osteoclast.

The role of TNF-α in the etiology of cherubism is under debate and could be different between murine and human cherubism. Mukai et al. showed that monocytes derived from heterozygous KI mice are sensitive to TNF-α and can differentiate into OCs independently of RANKL.^24^ However, the ability of TNF-α to induce OC differentiation of cells from patients with cherubism was not tested before. In order to evaluate the effect of TNF-α on OC differentiation using PBMCs from whole blood samples, we used the same conditions as in the differentiation experiment described above, but with the exception, that RANKL was substituted by TNF-α (100 ng/mL). Addition of TNF-α to PBMC cultures from the healthy controls failed to induce osteoclastogenesis, yielding only a few small multinucleated cells with low nuclei numbers (Figure 3). However, compared to the control samples, TNF-α induced a profound increase in the number of multinucleated cells (Figure 3A and B), nuclei in OCs (Figure 3C and D) and area of multinucleated cells (Figure 3E and F) in the cultures of PBMCs from either symptomatic or asymptomatic cherubism mutation carriers. Representative images are presented in Figure 3G. The statistical differences are summarized in Table 2.

**Figure 4:**
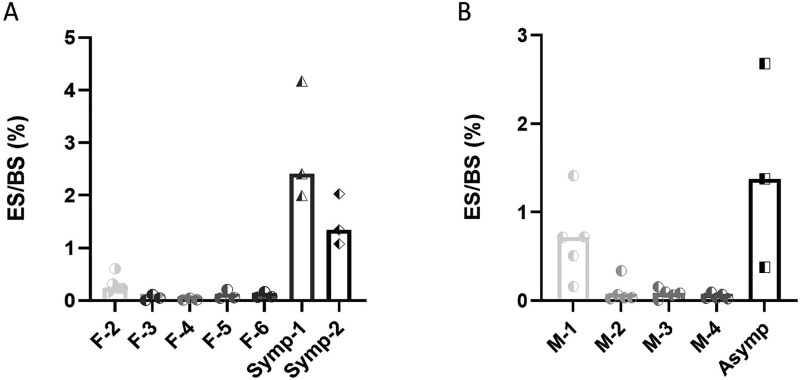
Osteoclasts from PBMCs of cherubism patients, driven by TNF-α, resorb more bone than healthy controls. Human PBMCs were cultured in a differentiation medium in the presence of M-CSF (25 ng/mL) and TNF-α (100 ng/mL) up to 14 d. (A and B) The percentage eroded surface (ES) per total bone surface. Data are median of *n* = 3. F, M, Symp and Asymp refer to female, male, symptomatic and asymptomatic, respectively. Groups and treatments were compared using multivariate generalized linear mixed effects model. *p*-values that are presented are based on comparisons between carriers (Symp-1 and Symp-2 for females or Asymp for males) vs age and gender matched controls.

To determine if formation of numerous large OCs by TNF-α is in correlation with enhanced bone resorption by these cells, we compared the bone resorption area in cultures of OCs derived from PBMCs of the two symptomatic and the asymptomatic cherubism mutation carriers. For this purpose, we used the same conditions as for the bone resorption assay described above with the exception that RANKL was substituted by TNF-α. The OCs generated from symptomatic as well as asymptomatic cherubism mutation carriers resorbed more bone when compared to the controls (Figure 4A and B).

**Figure 5:**
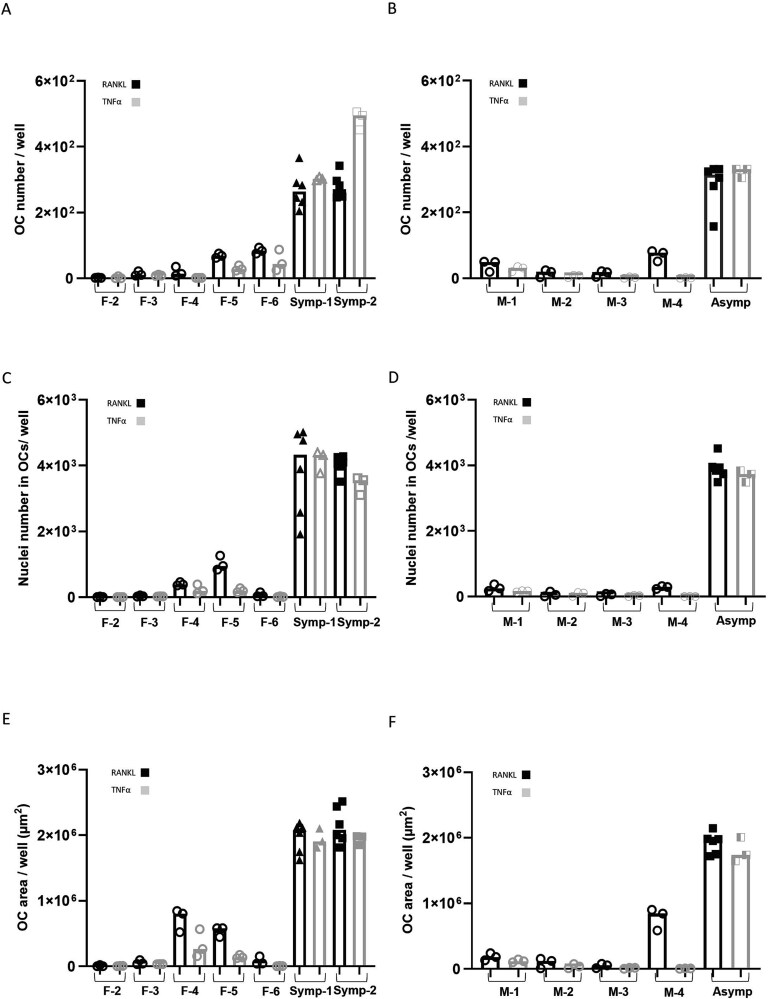
Effect of RANKL and TNF-α on OC differentiation from PBMCs of cherubism carriers and matched healthy controls. Differentiation data from Figures 1 and 4 are present as a comparison between RANKL and TNF-α treatment on OCs formation. (A and B) Total number of OCs, (C and D) total number of nuclei within OCs, and (E and F) Total surface area of OCs (G and H). Female samples (A, C, and E) and male samples (B, D, and F). Groups and treatments were compared using multivariate generalized linear mixed effects model. F, M, Symp and Asymp refer to female, male, symptomatic and asymptomatic, respectively.

**Figure 6:**
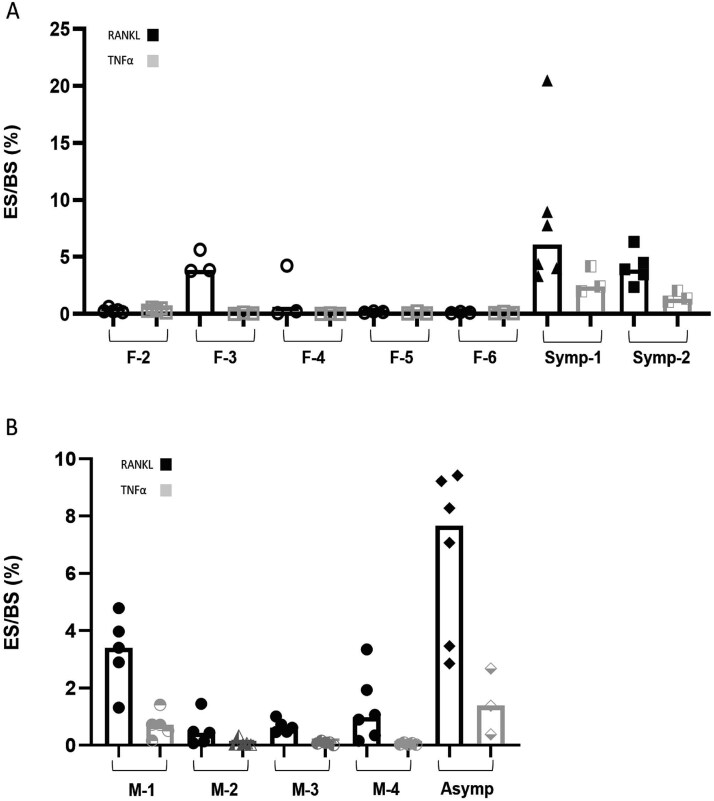
Effect of RANKL and TNF-α on OC activity from PBMCs of cherubism patients and controls. Resorption data from Figures 3 and 5 are present as a comparison between RANKL and TNF-α treatment on OCs activity. The percentage eroded surface (ES) per total bone surface (BS). Female sample (A) and male samples (B). F, M, Symp and Asymp refer to female, male, symptomatic and asymptomatic, respectively.

We then sought to determine if the effect of TNF-α addition on the differentiation and function of OCs from cherubism mutation carriers is similar to the effects of RANKL addition. To further illustrate the relative impact of RANKL and TNF-α on OC formation and resorption, we re-analyzed the primary data presented in Figures 1-4 and summarize this comparison in Figures 5 and 6. These figures provide an integrated statistical view of the differential effects of each cytokine on OC parameters derived from the same experimental dataset.

**Table 2:** Statistical comparison between OCs parameters of cherubism mutation-carriers vs matched healthy controls after TNF-α treatment.^a^

	**Symptomatic vs matched controls**		**Asymptomatic vs matched controls**	
**Parameter**	**Means ratio (95% CI)**	***p*-value**	**Means ratio (95% CI)**	***p*-value**
**OCs number/well**	32.5	<0.0001	42.7	0.0033
**Nuclei number/well**	65.2	0.0114	23.5 (6.2, 88.9)	<0.0001
**OC area/well**	42.9	0.0423	7 (2, 24.6)	0.0025
**Eroded surface/BS**	7.1 (2.5, 20)	0.0002	7.6 (1.2, 49.1)	0.0340

a If interaction term between treatment (RANKL/TNF-α) and group (carriers/controls) was not significant, results are identical to those of RANKL treatment. If the interaction term was significant, its effect was combined with that of RANKL to obtain the ratio for TNF-α.

Mean ratio-carriers vs matched control.

Abbreviation: CI, confidence interval.

Overall, across all experimental and control groups, the number of OCs, nuclei within OCs, OC area, and eroded surface per bone surface were lower in cultures treated with TNF-α compared to those treated with RANKL (Figures 5A-F and 6A and B, Table 3). Interestingly, despite better effects of RANKL on all other parameters between all groups, the multivariate analysis indicated that addition of TNF-α induced higher OC numbers then addition of RANKL in the cultures of PBMCs derived from the cherubism carriers (means ratio = 1.2, *p*-value = 0.0033 for asymptomatic; means ratio = 1.4, *p*-value < 0.0001 for symptomatic, these data are an extension to the findings presented in Table 3).

**Table 3:** Statistical comparison between OCs parameters of cherubism mutation carriers and matched healthy control-TNF-α vs RANKL treatment.

	**Symptomatic and matched controls**		**Asymptomatic and matched controls**	
**Parameter**	**Means ratio) 95% CI(**	***p*-value**	**Means ratio) 95% CI(**	***p*-value**
**OC number/well**	0.445 (0.44, 0.45)	<0.0001	0.23 (0.13, 0.43)	<0.0001
**Nuclei number/well**	0.38 (0.25, 0.59)	0.000012	0.36 (0.16, 0.82)	0.0142
**OC area/well**	0.31 (0.17, 0.61)	0.0005	0.16 (0.06, 0.44)	0.0004
**Eroded surface/BS**	0.18 (0.08, 0.39)	0.00002	0.16 (0.09, 0.30)	<0.0001

Mean ratio-TNFα vs RANKL treatments.

Abbreviation: CI, confidence interval.

## Discussion

In this case study, we evaluated the differentiation potential and aggressiveness of OCs derived from peripheral blood of two patients with clinical symptoms of cherubism to an asymptomatic carrier of the same SH3BP2, P418R mutation.

Several studies show a correlation between the differentiation and activity of OCs derived from PBMCs to skeletal pathology in humans. For example, the age and menopausal status correlate with the aggressiveness of OCs derived from peripheral blood.^22^ These OCs are likely reprogrammed as a result of the physiological condition that push toward a more aggressive OC behavior.^25^ Therefore, we sought to determine if the appearance of clinical symptoms of cherubism is correlated to differentiation and aggressive behavior of OCs in vitro. We show that upon stimulation with RANKL or TNF-α, monocytes from cherubism mutation carriers form more and bigger OCs and they contain a higher number of nuclei at an early time point compared to OCs derived from gender and age matched healthy controls. These results are in accordance with studies showing increased differentiation potential of OCs from the cherubism KI mice.^14^ The biggest differences between the SH3BP2 P418R mutation carriers to the healthy controls was the size, number of OCs, and nuclei number, and in some cases, OCs from patients were found to have more than 100 nuclei were observed. To the best of our knowledge, this was not reported for OCs generated from healthy controls. This observation suggests that the SH3BP2 P418R mutation promotes or fails to stop OC fusion. Others and we have shown that OC fusion is a cell heterotypic process involving “fusion founder” and “fusion follower” cells.^26^^,^^27^ We also showed that the size of OCs could be determined by the fusion potency of founder and follower cells.^28^ The cellular and molecular mechanisms underlying SH3BP2 regulation of OC fusion are still elusive, increased fusion potential of monocytes from the peripheral blood of cherubism patients could be attributed to either enhanced potential of fusion founder and fusion follower cells and remain to be evaluated.

Monocytes from the asymptomatic mutation carrier also formed higher numbers of big OCs than those from controls, and the results for the OCs formed by the symptomatic mutation carriers were similar. These data suggest that in humans, the cherubism mutations in SH3BP2 increase the differentiation potential of OC precursors but this differentiation potential does not correlate with the genetic penetrance of symptoms in cherubism patients.

In cherubism KI mice, TNF-α plays a pivotal role in driving systemic inflammation.^14^ It was shown that monocytes derived from the bone marrow of the KI mice can differentiate into OCs and resorb bone in vitro when cultured with TNF-α in a RANKL independent manner.^24^ It was also shown that bone resorbing OCs are formed independently of RANKL in vivo when cherubism KI mice are crossed with RANKL deficient mice.^29^ In humans, multinucleated cells in granulomas stain positive to TNF-α, but its expression is low.^17^^,^^24^ Our data show that the numbers, multinucleation, and size of OCs from carriers of the SH3BP2 P418R mutation are higher than OCs derived from healthy controls when cultured with TNF-α. Also, OCs derived from PBMCs of the carriers of the SH3BP2 P418R mutation resorb significantly more bone than the healthy controls when cultured with TNF-α. It should be noted that TNF-α is generally considered to act synergistically with RANKL and RANKL could be expressed by other cells within the culture system.^30^ Therefore, the effects of TNF-α in these experiments could not be interpretated independently of RANKL. However, when comparing the effects of TNF-α to RANKL, RANKL had stronger effects on OC differentiation and bone resorption. The only exception was multinucleated cell numbers that were higher in cells from carriers cultured with TNF-α. These differences could possibly derive from TNF-α effects on the generation of giant multinuclear cells and not necessarily OCs. The dominant effect of RANKL may explain why treatment of cherubism patients with the anti RANKL denusomab was successful,^23^ while treatment of patients with anti TNF-α drugs (adalimumab) had no apparent clinical improvement.^20^ Our data shows that OCs from both symptomatic and asymptomatic carriers of the P418R substitution in SH3BP2 are aggressive compared to healthy controls with no apparent difference between them. These data imply that although the P418R mutation in SH3BP2 confer an intrinsic OC aggressive phenotype it is not sufficient to dictate the appearance and severity of cherubism symptoms. Phenotypic heterogeneity among individuals who harbor the same SH3BP2 mutation appears to depend on how strongly external or physiological cues reinforce the TNF-α-centered autoinflammatory loop that underlies cherubism. Experimental and clinical data show that oral-microbial load and tooth-eruption-related micro-injury intensify TLR2/4-MYD88 signaling in mutant macrophages.^16^^,^^31^ In this pedigree, the father remains lesion-free despite carrying the same SH3BP2 mutation as his two affected daughters, and despite comparable vitamin-D status, oral hygiene, lack of smoking, medications, or documented major infections. These observations underscore that other “second-hit” elements—most plausibly eruption-related jaw micro-injury, subtle differences in innate-immune set-points, and stochastic or epigenetic modifiers—can determine whether the SH3BP2-primed autoinflammatory loop crosses the threshold for overt osteoclastogenesis in this pedigree. We cannot fully exclude the possibility that the father may have experienced a mild or subclinical form of cherubism earlier in life, which regressed spontaneously, as has been reported in some cases. However, available dental records and clinical history do not support this scenario, and there is no evidence of prior jaw pathology. Even if this possibility existed, our findings of enhanced OC fusion and bone resorption in his PBMC-derived OCs would still support the conclusion that the SH3BP2 mutation intrinsically primes OC hyperactivity, independent of overt disease manifestations.

## Institutional review board statement

The study was conducted in accordance with the Declaration of Helsinki, and approved by the Institutional Review Board of Soroka medical center Soroka medical center (No. 0166-17 SOR). Informed Consent Statement: Informed consent was obtained from all subjects involved in the study.

## Supplementary Material

supplementry_figure_ziaf148

## Data Availability

The data presented in this study are available on request from the corresponding author.
