# Supplementary figures and images for: Comparative analysis of osteoclast function in symptomatic and asymptomatic individuals with cherubism-causing SH3BP2 mutation
Source: JBMR Plus. 2025 Sep 9;9(10):ziaf148. doi: 10.1093/jbmrpl/ziaf148 (PMC12461697; doi:10.1093/jbmrpl/ziaf148)

**Supplementary figure**

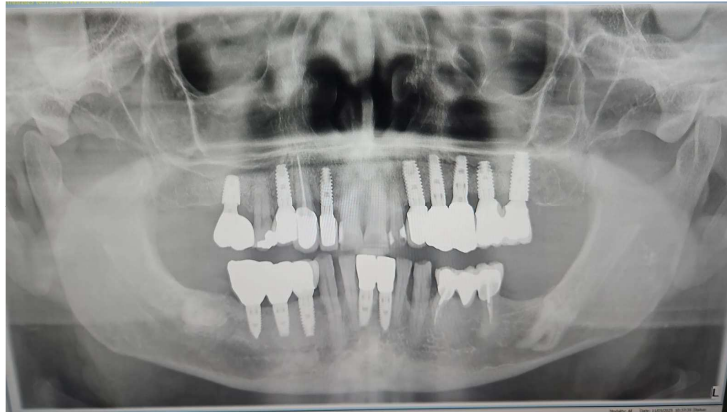

Radiographic dental imaging of the father, i.e. Asymptomatic

Supplement: supplementry_figure_ziaf148 [file supplementry_figure_ziaf148.pdf]
